# Isolation and molecular characterization of partial FSH and LH receptor genes in Arabian camels (*Camelus dromedarius)*

**Published:** 2015-06

**Authors:** Saber Jelokhani-Niaraki, Mojtaba Tahmoorespur, Morteza Bitaraf-Sani

**Affiliations:** Department of Animal Science, Faculty of Agriculture, Ferdowsi University of Mashhad, Iran

**Keywords:** FSHR, LHR, Sequence, Camel

## Abstract

Very little is known about *LHR *and *FSHR *genes of domestic dromedary camels. The main objective of this study was to determine and analyze partial genomic regions of *FSHR *and *LHR *genes in dromedary camels for the first time. To this end, a total of50 DNA samples belonging to dromedary camels raised in Iran were sent for sequencing (25 samples of each gene). We compared the nucleotide sequences of *Camelus dromedarius *with corresponding sequences of previously published *FSHR *and *LHR *genes in bactrian camels and other species. According to the data, the same nucleotide variation was identified in both regions of the two camel species. The alignment of deduced protein sequences of the two different species revealed an amino acid variation at the *FSHR *region. No evidence of amino acid variation was observed, however, in *LHR *sequences. Phylogenetic analysis indicated that both camel species had a close relationship and clustered together in a separate branch. This was further confirmed by genetic distance values illustrating significant sequence identity between *Camelus dromedarius *and *Camelus bactrianus*. Interestingly, sequence comparisons revealed heterozygote patterns in *FSHR *sequences isolated from dromedary camels of Iran. In comparison to other species, this camel contains three amino acid substitutions at 5, 67, and 105 positions in the *FSHR *coding region. These positions are found exclusively in camels and can be considered as species specific. The results of our study can be used for hormone functionality research (*FSHR *and *LHR*) as well as reproduction-linked polymorphisms and breeding programs.

## INTRODUCTION

The two gonadotropins, follicle-stimulating hormone (*FSH*) and luteinizing hormone (*LH*) are complex heterodimer glycoproteins, composed of a common alphasubunit and a hormone specific beta subunit. The subunits bind non-covalently to form a biologically active dimeric peptide hormone [1]. The effects of the two gonadotropins on ovarian development are mediated by their *FSH *and *LH *receptors. These receptors belong to the members of the GTP-binding protein super-family, characterized by the presence of a large extra-cellular domain [2, 3]. Multiple mRNA transcripts of *LHR *and *FSHR *have been identified in mammals, and it was found that the both relative abundance and size are specific to a given species and tissue [4].

In taxonomic ranks, the camel belongs to the Camelidae family, order Artiodactyla, Mammalian class [5, 6]. The Camelidae family is classified into two genera; new world camels and Camelus. The genus Camelus (old-world genus) includes two species: Camelus dromedarius or Arabian camel and the *Camelus bactrianus *or bactrian camel. The new world genus encompasses the Lama and the Vicugna [7]. The habitat of the Arabian camel consists of hot and dry regions such as Ethiopia, North Africa, Near East and West Central Asia. However, areas with colder weather tend to best suit Bactrian camels that occupy Mongolia, cold deserts of southern areas of Soviet Union, East Central Asia and China [8].

People of semi-dry and arid districts in Africa and Asia benefit from farming camels. The compatibility of camels with periods of extreme drought is characteristic, enabling them to reproduce in such climates [9, 10]; therefore, information on genetic variation levels in these species is much needed. Iranian Arabian and bactrian camel populations amount to about 150,000 and 100 heads, respectively. Bactrian camels typically populate the north-western part of Iran, the Ardebil province; however, their numbers are continually declining, causing them to be listed as endangered species [11].

One of the most important degradations in livestock resources is the lo ss of genetic diversity. Protecting genetic diversity in animal populations is crucially important in making genetic progress, and can lead to longer lasting production systems and access to various livestock products. In brief, genetic diversity creates a fundamental gene pool in order to develop a sustainable livestock production system [12].

In the present study, we determined the nucleotide and deduced amino acid sequences of partial *FSHR *and *LHR *genes for more than 20 camels from around Iran to investigate genetic relatedness. In addition, we carried out a phylogenetic analysis of these sequences to address the evolutionary relationship among the animal species.

## MATERIALS AND METHODS

**Sampling: **Blood samples of 25 healthy camels (twenty four Arabian and one bactrian) were randomly collected from a slaughterhouse in Yazd province where camels from different provinces in Iran including Kerman, Sistan & Baluchestan and Yazd are slaughtered. The samples were collected in tubes containing anticoagulant (EDTA), and transferred immediately to the Genomics’ Laboratory of the Department of Animal Sciences, Ferdowsi University of Mashhad (Khorasan Razavi Province, Iran). The tubes were placed at -20°C until used.

**DNA isolation and polymerase chain reaction (PCR): **Genomic DNA was extracted from the blood samples using NucleoSpin**® **Blood kit, (MACHEREY-NAGEL GmbH & Co. KG, Germany) according to the manufacturer’s guide. PCR primers were designed to amplify the given target regions of *FSHR *and *LHR *using OLIGO Primer Analysis Software (version 7.56) on the basis of *FSHR *and *LHR *sequences of bactrian camels. Nucleotide sequences of partial *FSHR *and *LHR *coding regions of bactrian camels are deposited in GenBank (accession No. GU990799 and GU301749,respectively). Genomic regions were amplified using standard methods and specific primer combinations in PCR. Primer sequences were as follows: Forward *LHR *(5′-CCT GAC CAG TCG CTA TAA ACT G-3′), Reveres *LHR *(5′-CCA GTA ACA CCT TAG AGT TGG T-3′), Forward *FSHR *(5′-TCC ACA CCA AAA GCC AGT ACC A-3′) and Reverse *FSHR *(5′-CAT GCA GAG GAA GTC CGT GAA G-3′). Amplified PCR fragments of expected lengths were electrophoresised on 1.2% agarose gel and analyzed under a UV transilluminator. Primers were targeted to amplify 674 and 488 bp of the *LHR *and *FSHR *genes, respectively. PCR was carried out in 50 µl reaction mixture containing 5 µl 10× reaction buffer, 1 µl mixed dNTPs (10 mM each), 1.25 U Taq DNA polymerase (CinnaGen, Tehran, Iran), 1.25 µl of each primer (10 pmol each), 2 µl DNA template (50 ng), 1.5 µl 50mM MgCl2, and 36.75 µµl ddH2O (double deionized water).

The following cycling conditions were applied for the amplification process: 94oC for 3 min; 35 cycles of 94oC for 45 sec, 58oC for 45 sec, 72oC for 50 sec, followed by 72oC for 5 min. Amplification conditions were the same for the two regions.

**Data analysis: **Obtained PCR products were analyzed on gel in order to confirm the correct fragment size of the product. Fragments were extracted and DNA products were purified from the agarose gel using Bioneer methods (Bioneer Co. Korea). Confirmed amplicons were sequenced from two directions using Bioneer sequencing methods, Bioneer Inc. (Daejeon, South Korea). Percent identity was measured using the MegAlign project of the DNAStar software package (version5.1). Table 1 shows the description of sequences used in our study. The phylogenetic tree (with bootstrap values) was created using the CLUSTAL X (2.0) and shown by the NJPLOT program. Partial nucleotide sequences of *FSHR *and *LHR *coding regions of Iranian camels have been submitted to GenBank with accession no. JX028597, KC290926, KC425610, JX028598 and KJ408448. In this analysis, published sequences of 41 *FSHR *and *LHR *coding regions from the world’s different species were included and compared with corresponding sequences of the Iranian camels. The sequences of each gene were initially examined to edit frame-shifted or incomplete ambiguous sequences. To determine the degree of observed genetic diversity in FSHR and LHR proteins, multiple alignments and comparisons of the predicted amino acid sequences were performed.

## RESULTS AND DISCUSSION

Partial nucleotide sequences of *FSHR *and *LHR *protein coding regions in camels were determined from PCR amplicons by sequencing and found to be of the expectedsize. Multiple data analyses were carried out to evaluate the genetic resemblance and divergence between camels and other species. The aim of the present study was to sequence 24 samples from dromedary camels and one sample from a bactrian camel in Iran. In total, 50 DNA samples belonging to dromedary camels were sent for sequencing (25 samples of each gene).

**Table 1:** The description of *FSHR* and *LHR* coding sequences used for multiple alignments and phylogenetic analysis

**Serial ** **No.**	**Species**	**Country**	**Date**	**Accession ** **No.**
1	*Camelus * *dromedarius*	Iran	2012	JX028597
2	*Camelus * *dromedarius*	Iran	2012	JX028598
3	*Camelus * *dromedarius*	Iran	2012	KC290926
4	*Camelus * *bactrianus*	Iran	2012	KC425610
5	*Camelus * *bactrianus*	China	2009	GU301749
6	*Camelus * *bactrianus*	China	2010	GU990799
7	*Capra * *hircus*	China	2009	FJ755812
8	*Ceratotherium * *simum*	China	2009	GU301755
9	*Elaphurus * *davidianus*	China	2010	HQ826052
10	*Moschus * *moschiferus*	China	2010	HQ826053
11	*Ailuropoda * *melanoleuca*	China	2010	XM_002928296
12	*Bos * *taurus*	China	2007	EU148061
13	*Bubalus * *carabanensis*	China	2007	EU148060
14	*Ailuropoda * *melanoleuca*	China	2010	XM_002912442
15	*Capra * *hircus*	China	2008	EU847288
16	*Ceratotherium * *simum*	China	2010	GU990800
17	*Elaphurus * *davidianus*	China	2010	HQ825702
18	*Hippopotamus * *amphibius*	China	2010	GU990797
19	*Moschus * *moschiferus*	China	2010	HQ825703
20	*Delphinapterus * *leucas*	China	2010	HQ826054
21	*Sus * *scrofa*	unknown	1989	M29526
22	*Gorilla gorilla * *gorilla*	unknown	2012	XM_004029219
23	*Bubalus * *bubalis*	India	2007	EU016216
24	*Macaca * *fascicularis*	Indonesia	2006	AM231185
25	*Canis * *lupus*	Unknown	2011	XM_538488
26	*Loxodonta * *africana*	USA	Unknown	XM_003417612
27	*Papio * *anubis*	USA	2012	XM_003908644
28	*Pongo * *abelii*	USA	2012	XM_002812041
29	*Pongo * *abelii*	USA	Unknown	XM_002812040
30	*Oryctolagus * *cuniculus*	unknown	2010	XM_002709875
31	*Ovis * *aries*	unknown	1996	L36329
32	*Canis * *lupus*	Brazil	2001	AF389885
33	*Homo * *sapiens*	Unknown	Unknown	M65085
34	*Macropus * *eugenii*	Australia	2002	AY082002
35	*Equus * *caballus*	Unknown	Unknown	NM_001164013
36	*Felis * *catus*	Unknown	Unknown	NM_001048014
37	*Mus * *musculus*	Unknown	Unknown	NM_013523
38	*Oryctolagus * *cuniculus*	Unknown	Unknown	XM_002709718
39	*Ovis * *aries*	Unknown	Unknown	NM_001009289
40	*Pan * *troglodytes*	Unknown	Unknown	XM_003309006
41	*Equus * *asinus*	France	1996	U73659
42	*Equus * *caballus*	France	2003	AY464091
43	*Bos * *taurus*	Japan	2002	AF491303
44	*Bubalus * *bubalis*	India	2006	DQ858168
45	*Homo * *sapiens*	unknown	1990	M63108

**Figure 1:**
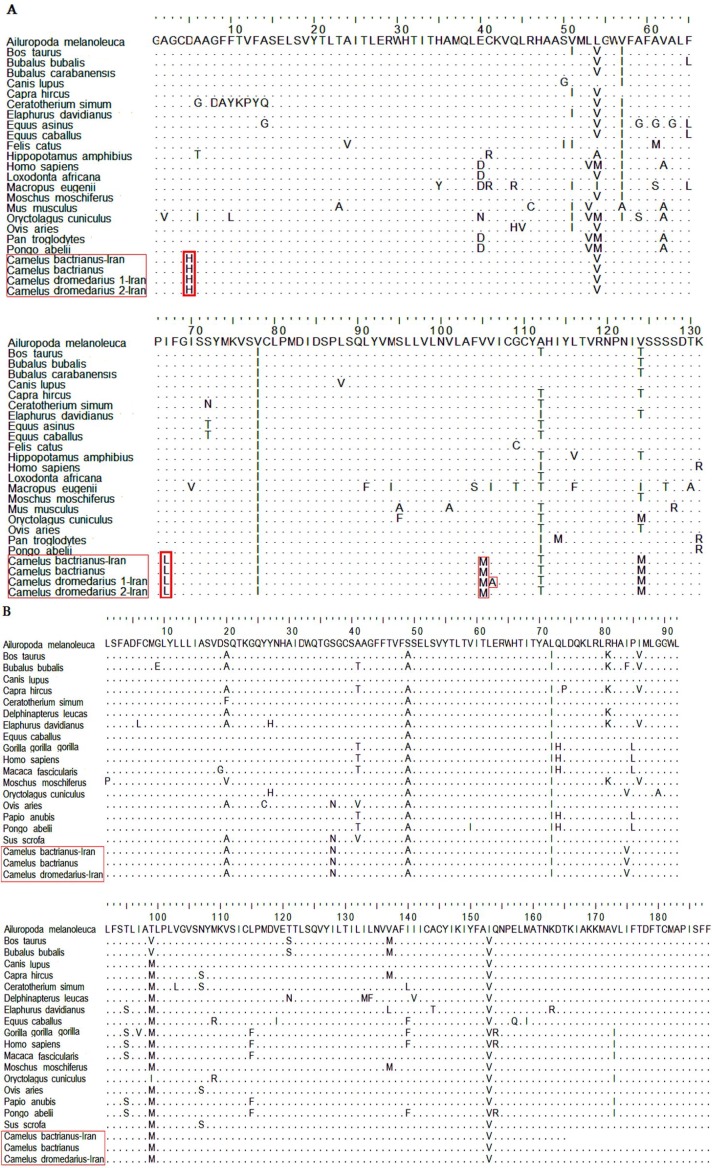
Alignment of partial amino acid sequences of *FSHR* (A) and *LHR* (B) region. Specific residues are marked with a red box. Dot (.) indicates sequence identity

**Figure 2:**
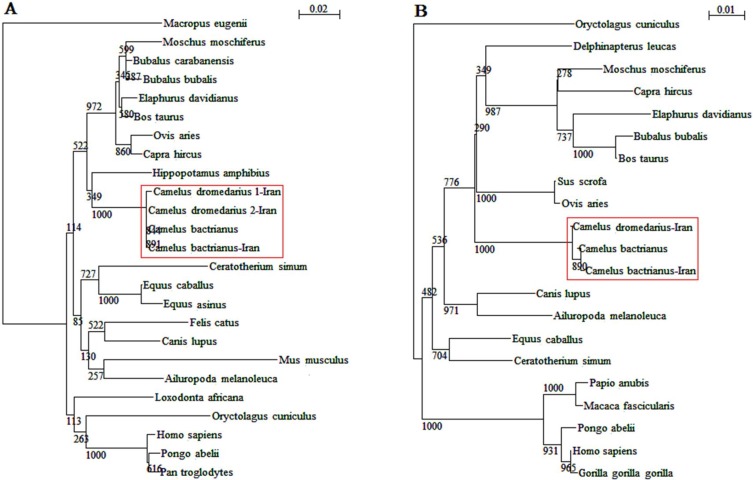
Phylogenetic tree constructed on the basis of partial sequences of *FSHR* (A) and *LHR* (B) region

**Figure 3:**
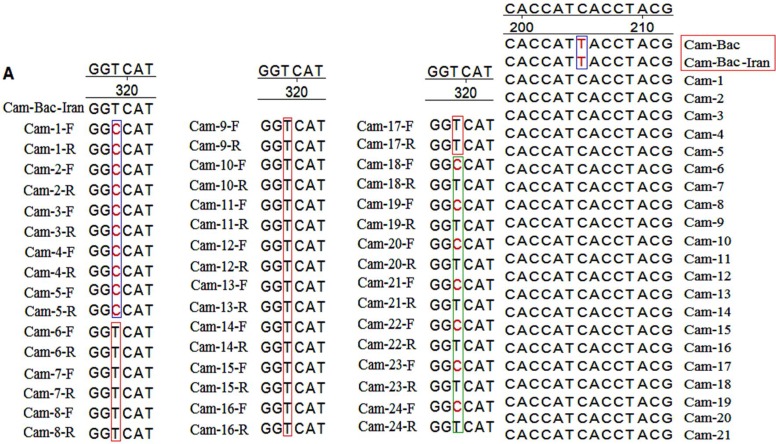
(A) The heterozygote and homozygote positions on *FSHR* sequences in examined dromedary camels of Iran. The letters F and R are abbreviated for forward and reverse sequence. (B) The position of single nucleotide polymorphism (SNP) found between two species Bactrian and Arabian camel in *LHR* region

Genetic distance values illustrated significant sequence identity (approximately 99%) between *Camelus dromedarius *and *Camelus bactrianus*. Nucleotide sequence analysis of the *LHR *region demonstrated that for the bactrian camel, there was a nucleotide T at the 205 position, while the same analysis showed a nucleotide C at the mentioned position for the dromedary camels (Fig. 3B). The sequence analysis of the camels’ FSHR regions revealed a substitution at position 319 (T is replaced by C) of *Camelus dromedarius*1-Iran, leading to the amino acid replacement of Val→Ala (Fig. 1A). The *FSHR *region of the *Ceratotherium simum *species contains a heptapeptide which is unique to this species. The similarity of the *LHR *region of dromedary camels with other species was estimated to fall between 90.6 and 94.7 (data not shown), while the similarity between all compared species fell within the 88.7 to 99.8 range. The similarity between the dromedary camels’ *FSH *region and that of other species ranged from 82.6 to 94.5, while the similarity range in the FSHR region across all compared species was found to fall between 81.6 to 99. These variations were distributed uniformly along the genes across the species. Figure 2 shows a phylogenetic tree constructed based on the sequence alignment of the 21 genomes of the *LHR *region and the 25 genomes of the *FSHR *region which are distinctly divided into different lineages. As depicted in Figure 2A, the four dromedary camels clustered with the *Hippopotamus amphibious *species into a branch separate from other species types. Figure 2B demonstrates that camels and all other species examined in the present study originated from different geographical areas, and did not cluster in relatively similar lineages based on *LHR *sequences. The phylogenetic tree topology indicates an insignificant similarity between camels and all other species in terms of their *FSHR *and *LHR *coding regions.

In the recent years, molecular biology and bioinformatics tools have provided researchers with a plethora of mammalian genomic data. Advances in different fields of biology point out the need to develop fundamental studies on mammalian genomes. Detailed knowledge and the understanding of the molecular characteristics of came l genome can be used to monitor processes such as evolution, genetic diversity and origination, and to genotype camel breeds.

In our study, which was one of the first of its kind to report partial nucleotide and deduced amino acid sequences for dromedary camels, *FSHR *and *LHR *partial genes were isolated and sequenced. FSH and LH pituitary derived gonadotropins play an important role in the regulation of gametogenesis and the production of steroid hormones in the gonads. Gonadotropins belong to the cystine-knot family and are heterodimeric glycoproteins consisting of a common α-subunit, non-covalently linked to a hormone specific β-subunit which causes biological activity [13-15]. *FSH *regulates reproductive processes including gonadal functions and fertility. In order to dispatch its signal, *FSH *needs to be bound to its receptor (*FSHR*). Therefore, mechanisms determining *FSHR *levels and cell-specific expressions control both the quantity and the target of hormone responses. Transcriptions of the *FSHR *gene also support these processes [16].

*LHR *plays a pivotal role in the ovarian response to *LH *[17]. According to previous reports, the *LHR *coding gene in ovarian follicles of species such as sheep and cattle may have alternative splicing. *LHR *splice variants have been found with deletion in exon 10 and/or partial deletion in exon 11 [18-20]. However, the authors are aware of no previous study addressing the coding sequence of camel *FSHR *and *LHR*. In the present study, the polypeptide region 7 transmembrane receptor (7tm_1) (rhodopsin family) of proteins *FSHR *and *LHR *was determined only. According to previous works, the *FSHR *coding sequence in buffalos comprises of 2085 bp ORF, encoding a protein with 695 amino acids [21]. *LHR *is composed of a single polypeptide chain [22]. Mature *LHR *is a single polypeptide with a predicted molecular mass of 75 kDa, comprising of 674 amino acids [23, 24]. As expected, the comparison of amino acid sequence homologies of partial *FSHR *and *LHR *across the species revealed that the dromedary camel had a higher degree of homology to the bactrian camel than the other animal species (Figure 1A and B). Nucleotide sequence analysis indicated that *FSHR *sequences isolated from dromedary camels in Iran were heterozygote for one nucleotide. As figure 3A shows, there is a clear evidence of heterozygosity in this region. At nucleotide position 319, five dromedary camels were homozygote for nucleotide C, 12 were homozygote for nucleotide T and 7 were heterozygote for nucleotides C and T.

The bactrian camel under study showed 100% homology with the sequence of another bactrian registered in GenBank. The number of sequence differences exhibited by each of the species showed that camels had three amino acid substitutions at positions 5, 67, and 105 in the *FSHR *coding region (Fig. 1A). In comparison to other species in the *FSHR *amino acid region, amino acid D is replaced by H at position 5, I is replaced by L at position 67 and V is replaced by M at position 105 (Asp5 → His, Ile67→ Leu, Val105→ Met). These positions are found exclusively in camels and can be considered specific to this species. Reasons for minor variations in nucleotide and amino acid sequences of *FSHR *and *LHR *between bactrian and dromedary camels are unknown, and further studies are required to discover, follow and analyze them. Since mutations seem to be a significant source of genetic novelty, gaining knowledge on the rate of point mutation is crucially important. In an earlier study conducted on mut ation rates in mammalian genomes, the mutation rate is believed to vary many times among genes of a genome and among mammalian lineages [25]. Based on our analysis, *LHR *amino acid sequences of the dromedary camel are highly conserved (Fig. 1B). Contrary to this, the *FSHR *sequences were prone to residue alteration. Phylogenetic analyses of sequences may enhance our understanding of the molecular evolution of *FSHR *and *LHR *genes in camels. In this study, output trees of phylogenetic analyses further confirmed the findings of the homology analysis. As expected, both camel species clustered together into a separate branch on the basis of selected regions.

In conclusion, the analysis of partial *FSHR *and *LHR *genes in dromedary camels demonstrates their closest homology with the bactrian camel. Our work provides fundamental genomic information on dromedary camels and is the first step towards the development of genomic data for *FSHR *and *LHR *genes in this species. Such information can be used in future research on hormone functionality (*FSHR *and *LHR*), reproduction-linked polymorphisms and breeding programs.

## Notes

**Acknowledgements: **This work was financially supported by the Ferdowsi University of Mashhad. Authors are thankful to Mr. Golghandashti for his collaboration.
